# Deep Learning Assisted Detection of Abdominal Free Fluid in Morison's Pouch During Focused Assessment With Sonography in Trauma

**DOI:** 10.3389/fmed.2021.707437

**Published:** 2021-09-23

**Authors:** Chi-Yung Cheng, I-Min Chiu, Ming-Ya Hsu, Hsiu-Yung Pan, Chih-Min Tsai, Chun-Hung Richard Lin

**Affiliations:** ^1^Department of Emergency Medicine, Kaohsiung Chang Gung Memorial Hospital, Chang Gung University College of Medicine, Kaohsiung, Taiwan; ^2^Department of Computer Science and Engineering, National Sun Yat-sen University, Kaohsiung, Taiwan; ^3^Department of Pediatrics, Kaohsiung Chang Gung Memorial Hospital, Chang Gung University College of Medicine, Kaohsiung, Taiwan

**Keywords:** deep learning, FAST, Morison pouch, ascites, trauma, hemoperitoneum

## Abstract

**Background:** The use of focused assessment with sonography in trauma (FAST) enables clinicians to rapidly screen for injury at the bedsides of patients. Pre-hospital FAST improves diagnostic accuracy and streamlines patient care, leading to dispositions to appropriate treatment centers. In this study, we determine the accuracy of artificial intelligence model-assisted free-fluid detection in FAST examinations, and subsequently establish an automated feedback system, which can help inexperienced sonographers improve their interpretation ability and image acquisition skills.

**Methods:** This is a single-center study of patients admitted to the emergency room from January 2020 to March 2021. We collected 324 patient records for the training model, 36 patient records for validation, and another 36 patient records for testing. We balanced positive and negative Morison's pouch free-fluid detection groups in a 1:1 ratio. The deep learning (DL) model Residual Networks 50-Version 2 (ResNet50-V2) was used for training and validation.

**Results:** The accuracy, sensitivity, and specificity of the model performance for ascites prediction were 0.961, 0.976, and 0.947, respectively, in the validation set and 0.967, 0.985, and 0.913, respectively, in the test set. Regarding feedback prediction, the model correctly classified qualified and non-qualified images with an accuracy of 0.941 in both the validation and test sets.

**Conclusions:** The DL algorithm in ResNet50-V2 is able to detect free fluid in Morison's pouch with high accuracy. The automated feedback and instruction system could help inexperienced sonographers improve their interpretation ability and image acquisition skills.

## Introduction

Traumatic injury remains the leading cause of death among individuals younger than 45 years of age (1), with over 210,000 deaths per year in the last 5 years in the United States (2). A substantial proportion of such patients suffered from blunt abdominal trauma (3). Computed tomography had been the gold standard for diagnosing intra-abdominal or thoracic injuries. However, time delays and transportation out of the emergency department (ED) hinders the evaluation of hemodynamically unstable patients. The use of focused assessment with sonography in trauma (FAST) enabled clinicians to rapidly screen for injury at the bedsides of patients. Recent studies have shown that FAST plays a key role in trauma detection, changing the subsequent management of an appreciable number of patients. In addition, pre-hospital FAST improves diagnostic accuracy and streamlines patient care, leading to dispositions to appropriate treatment centers. It was previously demonstrated that after performing pre-hospital FAST, pre-hospital therapy and management can be altered for 30% of the patients, and patient disposition can occur in 22% of the cases being admitted to the ED (4–6). A major limitation of ultrasound is that it is operator-dependent, with training, experience, and inter-operator variability playing an important role (7).

Artificial intelligence (AI), a subfield of computer science, helps create systems that perform tasks in medicine, medically oriented human biology, and healthcare improvements. Machine learning (ML), a subfield of AI, outperforms traditional approaches to various diseases and clinical conditions, including diagnosis, quality of patient care, and prognosis of a disease (8–14). Compared with traditional approaches, ML procedures may have the ability to interact with non-linear and high-order effects in variable parameters. Owing to the nature of operator-dependent imaging modality in ultrasound, developing deep learning (DL) models that assess image quality and provide feedback to sonographers was considered to provide ultrasound with more intelligence. AI-assisted ultrasound is expected to minimize operator-dependent imaging modality, altering medical therapy and patient disposition in critical care units and pre-hospital care.

Owing to the amorphous nature of intra-abdominal free fluid, AI-assisted ascites detection remains a challenge. Our study aims to determine the accuracy of AI model-assisted free-fluid detection during FAST examination and subsequently establish an automated feedback system, which can help inexperienced sonographers improve their interpretation ability and image acquisition skills. Moreover, AI model-assisted real-time ultrasound could enhance the diagnostic performance of FAST when used by paramedics or during an emergency.

## Materials and Methods

### Study Setting and Variables

This is a single-center study of patients admitted to the ED at Kaohsiung Chang Gung Memorial Hospital, Taiwan. Abdominal ultrasound clips were taken for a variety of clinical conditions and saved in the emergency ultrasound image archive in an MPEG-4 format. These clips were taken by 10 certified attending emergency physicians using a time-motion ultrasound machine with a 5–2 MHz curved mechanical sector transducer. The study was approved by the IRB committee of the hospital (IRB number: 202001766B0C601)

For the training set, all patients aged >18 years who underwent abdominal ultrasounds in the ED from January 2020 to October 2020 were included. Because of the study's retrospective nature in this study period, informed consent from the subjects was not required. Ultrasound examinations were retrospectively reviewed and retrieved from the image database in the ED during the study period. We only retrieved examinations performed on the right upper abdominal quadrant for Morison's pouch scanning. Morison's pouch is the space that separates the liver from the right kidney, considered the lowest intra-abdominal area for detecting free fluid in the supine position.

For the validation and test sets, patients aged >18 years who underwent FAST study in the ED from November 2020 to March 2021 were included. Informed consent was obtained from all subjects involved during this study period prior to beginning the abdominal ultrasound examinations.

To compare the ascites interpretation between emergency medicine (EM) residents and model performance, 10 registered EM residents were recruited for the trial from hospital personnel. Each EM resident had received at least a 1-year training course in the ED. The result for the EM residents' ultrasound finding interpretation of the test set was compared to that of the DL model in terms of accuracy, sensitivity, and specificity.

### Data Pre-processing and Labeling

All collected ultrasound videos were first converted to still images at a rate of 10 frames per second with an initial size of 800 × 600 pixels. Subsequently, each image was reviewed by 4 ultrasound instructors in the hospital to determine whether it was positive or negative for free-fluid detection.

A feedback labeling for the standard Morison's pouch view was subsequently added during the image review process to implement the assisting system that enabled the operator to distinguish whether the current image was qualified to detect the Morison's pouch fluid. The qualified view was defined as the area between the liver and kidney, caudal edge of the liver, or right paracolic gutter area. The image was classified as a non-qualified view if less than one-third of the right kidney was observable.

After the review process, each image was classified into one of four classes: positive/qualified view, positive/non-qualified view, negative/qualified view, and negative/non-qualified view. All images were labeled by four qualified ultrasound instructors who were certified attending emergency physicians in Taiwan, and at least 3 out of 4 instructors had to agree on the classification of each image.

### Deep Learning Training Process

To avoid overfitting the model, all labeled images were cropped to a size of 400 × 400 pixels to remove unnecessary information such as the background grid and knobology settings information. Other image augmentation techniques, namely, random rotation, random zoom, and horizontal flip, were applied to the training set.

In this study, we used the DL model Residual Networks 50-Version 2 (ResNet50-V2) for training and validation (15). With limited clinical data, we obtained model weights of ResNet50-V2 from the ImageNet database (16) as a pre-trained model and performed transfer learning through a fine-tuning process during training. During transfer learning, we froze the model weights in convolution layers 1 to 3 and updated the weights in convolution layers 4 and 5 and in the fully connected neural network on the top during training. All the aforementioned deep neural networks were developed using Python 3.8 and TensorFlow version 2.4.1.

### Statistical Analysis

We balanced positive and negative free-fluid detection groups with a 1:1 ratio during model training, validation, and testing to prevent deviations due to imbalanced data. We collected 162 patient records with positive free-fluid detection and randomly extracted 162 patient records with negative findings from the image database for the training model. We also collected 18 patient records each from the positive and negative groups for validation and another 18 patient records each for testing. Each patient record contained 15–35 second long ultrasound videos. During the DL training process, we tried to optimize the model to achieve the best performance in the validation set. The final performance was evaluated in the test set as an external validation.

The performance of the model for fluid detection was assessed in terms of accuracy, sensitivity, and specificity. The performance of the feedback was evaluated based on accuracy. In addition, from a clinical perspective, sensitivity is considered more relevant than specificity because it shows how accurately the intra-abdominal free fluid was identified; thus, the level of sensitivity was prioritized over specificity during training and validation.

For a real-time ultrasound assisting system, predicting a class for every input image might be difficult. Alternatively, generating a prediction every 10 frames is more feasible in clinical practice. Consequently, we also considered another evaluation strategy for model performance on ascites prediction in both validation and test sets, which employed a majority-voting scheme for consecutive images in a 1-s window, where the majority of the image class predictions were taken in that specific time frame.

In this study, continuous variables were presented as mean ± standard deviation. Dichotomous data were presented as numbers (percentages). Categorical variables were analyzed using the chi-square test, and continuous variables were analyzed using the independent sample *t*-test. All statistical analyses were performed using SPSS 26.

## Results

**Figure 1:**
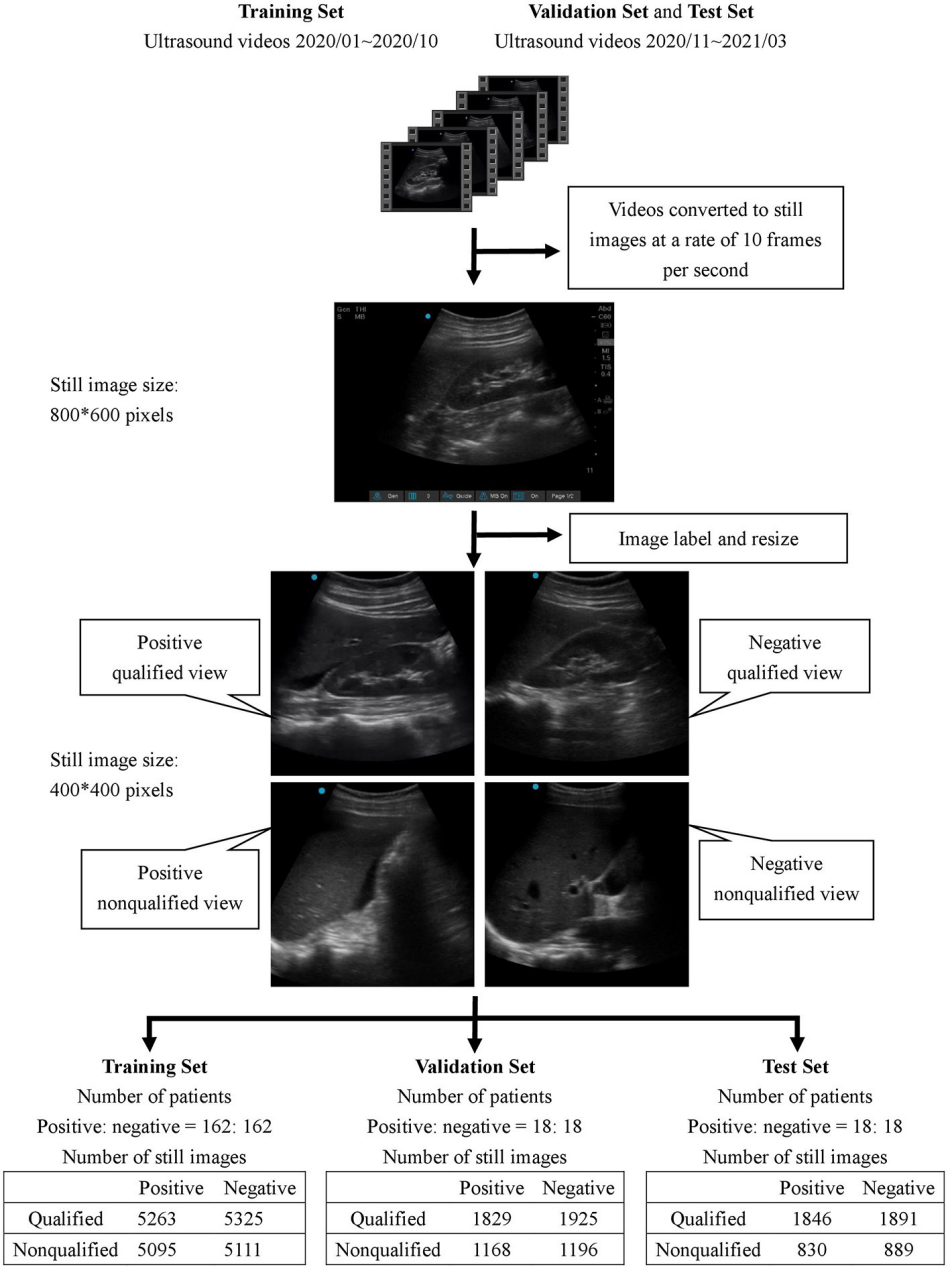
Flowchart of dataset construction.

The demographic characteristics of the patients included in the study are listed in Table 1. There was no significant difference in age, sex, body mass index, and underlying diseases between the training set, validation set, and test set. For training, validation, and testing, 10,794, 6,118, and 5,456 images were, respectively, included. In the validation set, there were 3,121 negative and 2,997 positive ascites images. Among them, 3,750 images were labeled as qualified images. In the 2,997 positive ascites images, there were 1,488 frames with mild ascites, 774 with moderate ascites, and 735 with massive ascites. In the test set, there were 2,780 positive and 2,676 negative ascites images. Among them, 3,337 images were labeled as qualified images. In the 2,676 positive ascites images, there were 1,332 frames with mild ascites, 682 with moderate ascites, and 662 with massive ascites. Figure 1 depicts a flowchart of the dataset construction process.

**Table 1:** Demographic data of patients.

	**Training set**	**Validation set**	**Test set**	***P* value**
	**(*n* = 324)**	**(*n* = 36)**	**(*n* = 36)**	
**Demographic characteristics**				
Age, years, mean ± SD	59.7 ± 17.2	61.1 ± 15.9	60.5 ± 19.2	0.889
Sex, male, *n* (%)	174 (53.7)	19 (52.8)	20 (55.6)	0.981
BH, mean ± SD	162.2 ± 8.8	161.8 ± 9.8	163.4 ± 8.5	0.731
BW, mean ± SD	60.9 ± 13.1	62.6 ± 14.2	62.3 ± 12.0	0.661
BMI (kg/m^2^), mean ± SD	23.3 ± 4.4	23.6 ± 4.3	23.4 ± 4.1	0.968
**Underlying disease**				
Heart failure, *n* (%)	19 (5.9)	3 (8.3)	2 (5.6)	0.779
Chronic kidney disease, *n* (%)	57 (17.6)	6 (16.7)	5 (13.9)	0.906
Liver cirrhosis, *n* (%)	40 (12.3)	5 (13.9)	6 (16.7)	0.840
Malignancy, *n* (%)	54 (16.7)	4 (11.1)	8 (13.9)	0.699

*BH, body height; BW, body weight; BMI, body mass index*.

To prevent the model from overfitting during training, we added a batch normalization layer and a dropout layer on top of the last fully connected neural layer of the final developed model. We used the Adam algorithm as an optimizer with an initial learning rate of 2 × 10^−6^ and adjusted the class weight to favor prediction over positive ascites for clinical priority purposes. The model was trained for 100 epochs, and the best model weights during training were saved and evaluated in the validation and test datasets.

**Figure 2:**
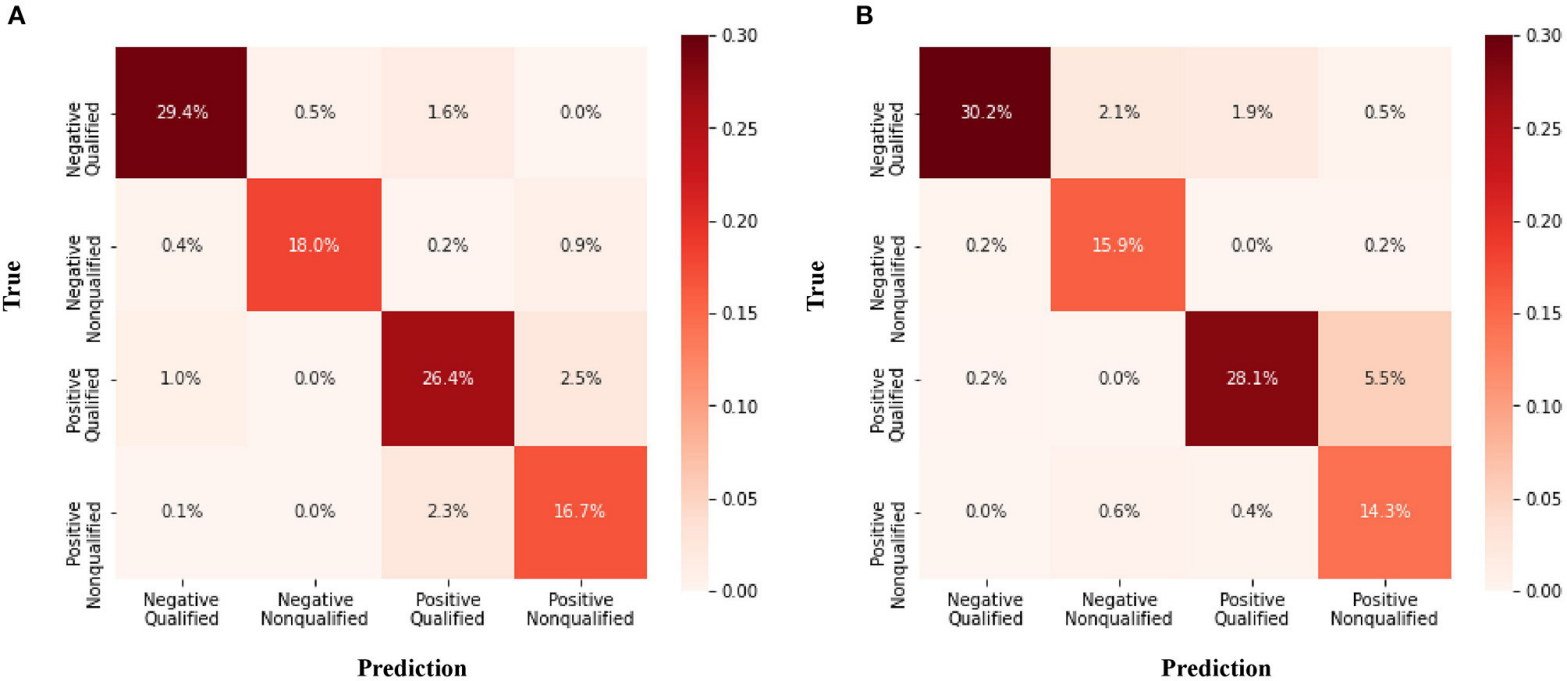
Confusion matrix for the four class prediction results. **(A)** Validation set **(B)** Test set.

The confusion matrix for the four class prediction results in the validation and test sets are shown in Figure 2. The accuracy, sensitivity, and specificity of the model performance for ascites prediction were 0.961, 0.976, and 0.947, respectively, in the validation set and 0.967, 0.985, and 0.913, respectively, in the test set (Table 2). For ascites prediction in the EM resident group, the accuracy, sensitivity, and specificity were 0.966, 0.989, and 0.943, respectively (Table 3). The result for human interpretation was not significantly different compared with the DL model (*p* = 0.570). Regarding feedback prediction, the model correctly classified qualified and non-qualified images with an accuracy of 0.941 in both the validation and test sets (Table 2).

**Table 2:** Model performance for ascites and image location feedback prediction.

	**Validation set**	**Test set**
**By frame**	**(*****n*** **= 6,118)**	**(*****n*** **= 5,456)**
*Ascites prediction*		
Accuracy	0.961	0.967
Sensitivity	0.976	0.985
PPV	0.947	0.949
Specificity	0.947	0.913
*Feedback prediction*		
Accuracy	0.941	0.941
**By 1-s majority voting**	**(*****n*** **= 309)**	**(*****n*** **= 500)**
*Ascites prediction*		
Accuracy	0.997	0.998
PPV	0.993	0.996
Sensitivity	1	1
Specificity	0.994	0.996

*PPV, Positive predictive value*.

**Table 3:** Comparison of resident doctor and model performance for ascites interpretation.

	**Resident physician (n=10)**	**ResNet50-V2 model**	***P* value**
**By frame**	**(*****n*** **= 5,456)**		
Accuracy	0.966	0.967	0.570
Sensitivity	0.989	0.985	
Specificity	0.943	0.913	
**By 1-s majority voting**	**(*****n*** **= 500)**		
Accuracy	0.986	0.998	0.033^*^
Sensitivity	1	1	
Specificity	0.972	0.996	

* *p < 0.05*.

By using the aforementioned majority-voting scheme for evaluation, the model was able to identify every ascites clip in both the validation and test sets, while it misclassified only two negative ascites frames: one into the positive class in the validation set and one into the positive class in the test set. The results of all the prediction performances are provided in Table 2. The accuracy, sensitivity, and specificity of the resident physician vs. model performance were, respectively, 0.986 vs. 0.998, 1 vs. 1, and 0.972 vs. 0.996 (*p* = 0.001).

## Discussion

In our study, we detected abdominal free fluid and predicted the location of the Morison pouch using only a single frame of the ultrasound image. ResNet50-V2 was able to detect the abdominal free fluid of the Morison pouch with accuracy, sensitivity, and specificity values of 96.1, 97.6, and 94.7%, respectively, in the validation set and 96.7, 98.5, and 91.3%, respectively, in the test set. The result of ResNet50-V2 performance was non-inferior to the EM resident interpretation. By using the majority-voting scheme for consecutive images in a 1-s window, the DL model was able to reach 100% sensitivity, and the specificity was significantly better than the EM resident interpretation. Previous studies demonstrated that FAST examinations with human interpretation for intra-peritoneal free-fluid detection have a sensitivity ranging from 61.3 to 100% and specificity ranging from 94 to 100% for blunt abdominal trauma (17–20). The sensitivity of the ultrasound examination (28–100%) was considered insufficient for it to be used alone in determining operative intervention for penetrating torso trauma (21–23). Although initially developed for the evaluation of trauma patients, FAST examination can also be used in non-trauma patients to narrow down differential diagnoses, change patient disposition, expedite consultation, avoid unnecessary procedures, and alter imaging needs (24). However, studies of sensitivity and specificity are limited owing to the large variety of etiologies in non-trauma patients.

Applications of DL in medical ultrasound analysis include anatomical applications, diagnosis tasks (classification, segmentation, detection), and clinical tasks (computer-aided diagnosis, biometric measurements, image-guided interventions) (8). DL models have been used to detect different anatomical structures of human organs in medical analyses, including the brain (9), heart (10), thyroid (11), breast (12), liver (13), and prostate (14). Recognized as one of the most popular deep architectures, the convolution neural network (CNN) has been applied to various tasks, such as image classification, object detection, and target segmentation (9, 25). Our study shows that a computer program developed incorporating ResNet50-V2 could aid in the detection of free fluids in FAST examination, with the results obtained on par with the interpretations of a medical doctor.

In various ultrasound protocols, performance plateaus occur at different points for image interpretation and quality (26). In addition, physicians acquire the ability to interpret FAST images quicker than they acquire the technical skills required to perform the examination (27). Focusing on the acquisition of images for FAST examination, Jang et al. (27) found that the ultrasound technique continued to improve even after 75 examinations. Blehar et al. demonstrated that the learning curve in image quality for FAST examination improved even after 200 examinations (26). In our model, the accuracy of locating Morison's pouch reached 94.05 and 91.35% in the validation and test sets, respectively. The automated feedback and instruction system is believed to assist inexperienced sonographers improve their interpretation ability and image acquisition skills.

Free-fluid detection by ultrasound could be used in both trauma and non-trauma patients and could have a broad impact on patient care across a wide range of medical settings (28). In addition, AI model-assisted real-time ultrasound could enhance the diagnostic performance of FAST when used by paramedics or during an emergency. Using FAST examination in the pre-hospital stage can significantly improve the outcomes of blunt abdominal trauma (5). However, to obtain the benefits of AI model-assisted FAST in-patient care, additional large-scale studies should assess the performance of free-fluid detection for different trauma and non-trauma etiologies.

The limitations in our study are discussed below. First, we trained using only perihepatic views in our study. In certain conditions, a single Morison's pouch view was employed because the right upper quadrant was considered the primary area where free fluid is initially seen and the most sensitive for free-fluid assessment (29, 30). However, a multiple-view FAST examination was recommended to increase sensitivity (31). Second, we tested our model with a single-frame ultrasound image and 1-s majority voting, which may have led to variable sensitivity and specificity. The purpose of free-fluid detection should be for identification using serial video clips. Third, the development of automated feedback and instructions by AI model-assisted free-fluid detection remains a challenge. For the classification of qualified or non-qualified view, the binary classifications in our study potentially caused a loss of continuous information. The feasibility of a real-time AI model-assisted system should be tested in future studies, including ultrasound acquisition software, real-time feedback speed, and indication for proper probe location. Finally, the performance of free-fluid detection for different etiologies should also be considered. Future studies should also include a separate assessment of the test performance for different free fluid etiologies, such as blunt trauma, penetrating trauma, and various non-trauma etiologies.

AI model-assisted real-time ultrasound could enhance the diagnostic performance of FAST when used by paramedics or during an emergency. The DL algorithm with ResNet50-V2, used in our study, was able to detect free fluid in Morison's pouch with accuracies reaching 94.05 and 91.35% in the validation and test sets, respectively. By using the majority-voting scheme for consecutive images in a 1-s window, the DL model was able to reach 100% sensitivity, and the specificity was significantly better than the EM resident interpretation. In the future, AI-assisted ultrasound will minimize operator-dependent imaging modality and alter medical therapy and disposition in critical care units and prehospital care.

## Data Availability Statement

The original contributions presented in the study are included in the article/supplementary material, further inquiries can be directed to the corresponding author/s.

## Ethics Statement

The studies involving human participants were reviewed and approved by the Institutional Review Board of Chang Gung Medical Foundation (protocol code: 202001766B0C601). The patients/participants provided their written informed consent to participate in this study.

## Author Contributions

C-YC conceived the research, performed the analyses, and wrote the manuscript. H-YP and C-MT contributed to data collection and measurements. M-YH was involved mainly in data analysis and quality management. I-MC and C-HL provided overall supervision, edited the manuscript, and undertook the responsibility of submitting the manuscript for publication. All authors read and approved the final manuscript.

## Funding

This research was funded by the Ministry of Science and Technology research grant in Taiwan (MOST 110-2314-B-182A-009).

## Conflict of Interest

The authors declare that the research was conducted in the absence of any commercial or financial relationships that could be construed as a potential conflict of interest.

## Publisher's Note

All claims expressed in this article are solely those of the authors and do not necessarily represent those of their affiliated organizations, or those of the publisher, the editors and the reviewers. Any product that may be evaluated in this article, or claim that may be made by its manufacturer, is not guaranteed or endorsed by the publisher.
